# Peak Risk of Recurrence Occurs during the First Two Years after a Pancreatectomy in Patients Receiving Neoadjuvant FOLFIRINOX

**DOI:** 10.3390/cancers15215151

**Published:** 2023-10-26

**Authors:** Marie-Sophie Alfano, Jonathan Garnier, Anaïs Palen, Jacques Ewald, Gilles Piana, Flora Poizat, Emmanuel Mitry, Jean-Robert Delpero, Olivier Turrini

**Affiliations:** 1 Department of Surgical Oncology, Institut Paoli-Calmettes, 13009 Marseille, France; alfanom@ipc.unicancer.fr (M.-S.A.);; 2 Department of Radiology, Institut Paoli-Calmettes, 13009 Marseille, France; 3 Department of Pathology, Institut Paoli-Calmettes, 13009 Marseille, France; 4 Department of Oncology, Institut Paoli-Calmettes, 13009 Marseille, France; 5 Faculté de Médecine, Aix-Marseille University, 13005 Marseille, France

**Keywords:** FOLFIRINOX, borderline, pancreatic adenocarcinoma, recurrence, survival, follow-up

## Abstract

**Simple Summary:**

No codified/systematic surveillance program exists for borderline/locally advanced pancreatic ductal carcinoma treated with neoadjuvant FOLFIRINOX and secondary resection. This study aimed to determine the trend of recurrence in patients who were managed using such a treatment strategy. From 2010, 101 patients received FOLFIRINOX and underwent a pancreatectomy, in a minimum follow-up of 5 years. The risk of recurrence in patients with T1/T2 N0 R0 was the lowest (19%), and all recurrences occurred during the first two postoperative years. The peak risk of recurrence for the entire population was observed during the first two postoperative years. The probability of survival decreased until the second year and rebounded to 100% permanently, after the ninth postoperative year. Close monitoring is needed at reduced intervals during the first 2 years following a pancreatectomy and should be extended to later than 5 years for those with unfavorable pathological results.

**Abstract:**

No codified/systematic surveillance program exists for borderline/locally advanced pancreatic ductal carcinoma treated with neoadjuvant FOLFIRINOX and a secondary resection. This study aimed to determine the trend of recurrence in patients who were managed using such a treatment strategy. From 2010, 101 patients received FOLFIRINOX and underwent a pancreatectomy, in a minimum follow-up of 5 years. Seventy-one patients (70%, R group) were diagnosed with recurrence after a median follow-up of 11 months postsurgery. In the multivariable analysis, patients in the R-group had a higher rate of weight loss (*p* = 0.018), higher carbohydrate antigen (CA 19-9) serum levels at diagnosis (*p* = 0.012), T3/T4 stage (*p* = 0.017), and positive lymph nodes (*p* < 0.01) compared to patients who did not experience recurrence. The risk of recurrence in patients with T1/T2 N0 R0 was the lowest (19%), and all recurrences occurred during the first two postoperative years. The peak risk of recurrence for the entire population was observed during the first two postoperative years. The probability of survival decreased until the second year and rebounded to 100% permanently, after the ninth postoperative year. Close monitoring is needed at reduced intervals during the first 2 years following a pancreatectomy and should be extended to later than 5 years for those with unfavorable pathological results.

## 1. Introduction

After the initial staging, approximately one-third of patients with nonmetastatic pancreatic ductal adenocarcinoma (PDAC) show major vascular invasion (i.e., portal vein/superior mesenteric vein, hepatic artery, celiac axis, and superior mesenteric artery) or biologically advanced disease (as evidenced by carbohydrate antigen [CA 19-9 > 500 kU/L]) [1,2]. In such patients, neoadjuvant treatment (gemcitabine or FOLFIRINOX) followed by resection significantly improves survival compared to upfront resection [3,4].

Some groups have advocated that the neoadjuvant chemotherapy/surgery therapeutic base can be optimized by adding neoadjuvant chemoradiation [5] and administering postoperative chemotherapy [6]. However, this ambitious strategy is possible only in a small proportion of fit patients. Thus, if most teams initiate the neoadjuvant treatment strategy, the modalities have not been standardized yet.

Once the therapeutic sequence, including surgery and adjuvant chemotherapy, chosen by the center is implemented, the surveillance period can be used to focus on detecting recurrence to allow appropriate treatment. Oncologic teams agree that patients should be monitored based on a combination of clinical examinations, CA 19-9 serum level, and computed tomography (CT)-scan. However, there is no consensus on the optimal frequency and duration of monitoring (≥5 years) [7] or its necessity [8]. BL/LA PDAC is an aggressive cancer, which explains why recurrences are frequent, especially early (before 5 years). Conversely, it is possible to consider the possibility of a cure within 5 years without any adverse events. Therefore, the monitoring of a patient is of interest because the earlier a recurrence is detected, the more effective the treatment to allow for new remission [9,10]. However, this approach requires significant cost [11] and a probable psychological impact on the patients, generating anxiety while awaiting each examination.

Our institution has more than 10 years of experience with neoadjuvant FOLFIRINOX and surgical treatment for patients with BL/LA PDAC. The aim of our study was to determine the annual risk of recurrence in patients who were managed by such a treatment strategy, and to establish an appropriate surveillance protocol and a discontinuation date when the risk becomes zero.

## 2. Materials and Methods

### 2.1. Ethical Statements, Study Design, and Population

The study protocol adhered to the tenets of the Declaration of Helsinki and was approved by the Institutional Review Board (IRB) at Institut Paoli Calmettes (PDAC-IPC 2023-018). We explored our prospectively maintained pancreatic surgery database (NCT02871336) and identified 179 consecutive patients who underwent a pancreatectomy after FOLFIRINOX induction from 1 January 2010 to 31 December 2017 (with a censored date of 1 January 2023). The requirement for informed consent was waived by the IRB owing to the retrospective nature of the study. Patients who survived at least 1 year after surgery (i.e., patients who died postoperatively or failed to thrive were excluded) and those for whom follow-up was a minimum of 5 years, were selected. We excluded patients who died before 1 year postsurgery (n = 3) to avoid the bias of the death due to postoperative complications or chemotherapy-induced complications because such patients would likely have been receiving adjuvant chemotherapy. Moreover, patients with disease recurrence before one year often exhibit questionably controlled disease before surgery and thus indication. Thus, in total, 101 patients were included in this study. This observational investigation was conducted according to the 2007 Strengthening the Reporting of Observational Studies in Epidemiology (STROBE) and the 2019 Strengthening the Reporting of Cohort Studies in Surgery (STROCSS).

### 2.2. Neoadjuvant Treatment, Surgery, and Postoperative Treatment

Patient selection, therapeutic strategy, and surgery for the neoadjuvant FOLFIRINOX/surgery sequence have been previously reported [12]. Briefly, all patients underwent an endoscopic ultrasound (EUS) combined with fine-needle aspiration (FNA). When the EUS failed more than three times in our institution, chemotherapy was delivered without proof if there was sufficient evidence for BL PDAC (anatomically, biologically with elevated CA 19-9, and with metabolism assessed by FDG-PET). Consequently, the entire patient population had a confirmed diagnosis of pancreatic adenocarcinoma at the final histopathological analysis. These patients were classified as having borderline (BL) or locally advanced (LA) disease [1] and necessarily underwent systemic treatment before surgery.

The criteria used to select patients were based on the ABC classification proposed by Isaji et al. [2] and on the vascular criteria of the National Comprehensive Cancer Network (NCCN) classification. After re-evaluation, surgery was considered according to the three criteria of anatomical reconstruction, disease response to neoadjuvant treatment, and patient clinical status. After receiving six cycles of neoadjuvant chemotherapy, patients were re-evaluated with the classic tripod, including clinical examination, CA 19-9, and CT-scan/MRI. If the performance status (PS) was >2, the CA 19-9 had regressed, and the scan showed a resectable tumor with no metastasis, we proceeded with surgery within a month from the last cycle. Depending on the complexity of the vascular reconstruction and patient tolerance of chemotherapy, we continued chemotherapy with a control every three cycles, for a maximum of 12 cycles. The combination of an acceptable PS, a reduced CA 19-9, and a reconstructable vascular involvement (veinous and arterial) at a timepoint of estimated response or stability of the disease, were necessary to discuss about surgery.

A pancreaticoduodenectomy (PD) was applied for the head tumor, including a standardized lymphadenectomy along the right side of the vascular structures (superior mesenteric artery, celiac axis) and the hepatoduodenal ligament. We performed PD without preservation of the pylorus. For tumors of the body and tail of the pancreas, a distal pancreatectomy and splenectomy with respective lymphadenectomy from the left side of the vascular structures were applied. In the case of the tumor being located in the center of the pancreas, a synchronous multiple PDAC, or a high risk of postoperative fistula, a total pancreatectomy with or without a splenectomy was considered to be required.

### 2.3. Follow-Up

Since there is no international consensus, all patients were followed up according to an institutional protocol. The monitoring rate was every 4 months during the first 2 years and every 6 months from the second to tenth postoperative years. Follow-up included clinical examination, serum CA 19-9 level evaluation, and a thoracoabdominal CT scan. Liver magnetic resonance imaging (MRI) and/or positron emission tomography (PET-CT) were performed in cases of suspicion of recurrence not confirmed by these three examinations (isolated elevation of CA 19-9 or doubtful image on the CT scan). The decision to use chemotherapy as the first treatment was made after multidisciplinary staff discussions, and biopsy-proven recurrence was not mandatory if there was biological or radiological evidence.

### 2.4. Study Variables

The variables evaluated were age, sex, the American Society of Anesthesiologists (ASA) score, body mass index (BMI), weight loss > 5% [13], head tumor location, back pain, biliary stenting, serum CA 19-9 level at diagnosis and after resolution of jaundice, number of FOLFIRINOX cycles administered, type of surgery (pancreaticoduodenectomy, total pancreatectomy, or left pancreatectomy), vascular resection, margins (resection margin < 1 mm was considered to be an involved margin [R1]) [14], node stage (N0 or N+), perineural invasion, and adjuvant treatment administration. Disease staging was performed according to the TNM classification of the American Joint Committee on Cancer (8th edition) [15]. No patients were lost to follow-up, and the censored date was 1 January 2023. The date and type (metastatic, local, or both) of recurrence were noted.

### 2.5. Endpoints

The primary endpoint of the study was the risk of recurrence in every postoperative year. The secondary endpoints were recurrence and probability of survival.

### 2.6. Statistical Analysis 

Data were analyzed using GraphPad Prism version 8 (GraphPad Software Inc., La Jolla, CA, USA). Categorical factors were compared using Fisher’s exact test or chi-squared test, and continuous variables were compared using the Student’s *t*-test. Multivariable analysis was performed using stepwise logistic regression, integrating factors identified in the univariable analysis at *p* < 0.1. Statistical significance was set at *p <* 0.05. The risk of recurrence and probability of survival were calculated for each year based on actual data and were therefore not estimated using the Kaplan-Meier method. 

## 3. Results

### 3.1. Patients’ Characteristics, Surgery, and Pathologic Findings

A total of 101 patients underwent resection after a median of six cycles of FOLFIRINOX (range 4–12). In this population, only 6% of patients had an LA PDAC at diagnosis. Most patients underwent a pancreaticoduodenectomy or total pancreatectomy (73%) with vascular resection (66%). Most patients had a T3/T4 tumor stage (66%), positive lymph nodes (61%), perineural invasion (77%), R0 resection (76%), or adjuvant chemotherapy (61%). All the data from the 101 patients are summarized in Table 1.

### 3.2. Recurrences 

Seventy-one patients (70%; R group) were diagnosed with recurrence after a median follow-up of 11 months (range 3–65) after surgery. Recurrences occurred in metastatic, local, or both in 78%, 11%, and 11% of patients, respectively. The data of patients with and without recurrence (NR group) are summarized in Table 2.

In multivariable analysis, patients in the R-group had a higher rate of weight loss at diagnosis (odds ratio [OR] = 1.297, 95% confidence interval [CI] 1.103; 1.783, *p* = 0.018); higher CA 19-9 serum level at diagnosis (OR = 3.42, 95% CI 1.31; 9.17, *p* = 0.012); T3/T4 tumor stage (OR = 3.30, 95% CI 1.25; 8.96, *p* = 0.017), and positive lymph nodes (OR = 3.85, 95% CI 1.46; 10.5, *p* < 0.01) than patients in the NR-group.

The number of FOLFIRINOX cycles and adjuvant chemotherapy administrations were not identified as factors influencing recurrence (margin resection status could not be integrated into the multivariate analysis because the number of events was too small to permit such an analysis). Of the patients, 66% received adjuvant chemotherapy with the FOLFIRINOX protocol and 33% presented with recurrence. This protocol was adapted in terms of the performance status of the patient. Concerning changes in the serum evaluation of CA 19-9, 89 patients (88%) had a decrease or stable serum evaluation of CA 19-9 after neoadjuvant chemotherapy, 62 of whom experienced recurrence. Conversely, 12 patients had an increase of CA19-9, 9 of whom experienced recurrence.

Chemoradiation therapy was administered to eight patients, seven of whom were classified as BL at diagnosis. This treatment was proposed in the context of clinical trials.

The risks of recurrence (latest recurrence) in patients with lymph node invasion or R1 status were 82% (65 postoperative months) and 92% (40 postoperative months), respectively. The only pathological combination that reduced the risk of recurrence was T1/T2 stage N0 and R0 (19%, *p* = 0.023) (Table 3); in such patients (n = 18), recurrence (n = 3) always occurred during the first two postoperative years.

**Figure 1:**
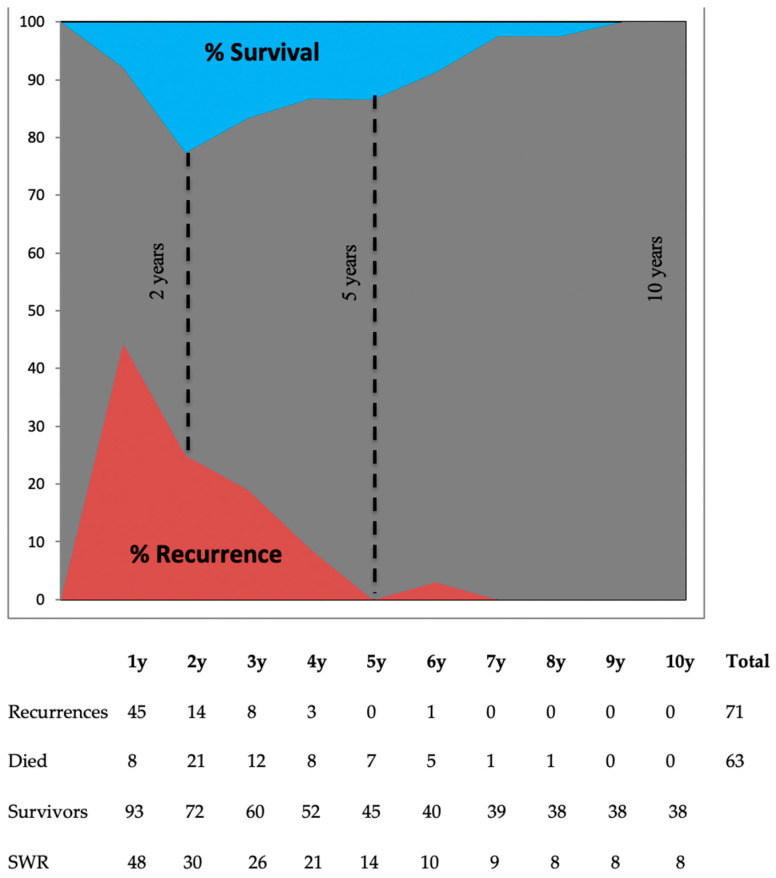
Probability of recurrence and survival for each postoperative year for the 101 patients. SWR: Survivor without progression.

The peak risk of recurrence for the entire population was during the first two postoperative years (44% at one year) and was nil from the seventh year of follow-up (Figure 1).

### 3.3. Survival

During the study period, 63 patients died of PDAC, corresponding to a survival rate of 38%. The real 1-, 3-, and 5-year survival rates of the 101 patients were 92%, 59%, and 38%, respectively. The probability of survival decreased until the second year and then rebounded to reach 100% permanently after the ninth postoperative year (Figure 1). Survivors without recurrence (SWR) included only eight of 38 total survivors from the 8th until the 10th year.

## 4. Discussion

The present study provides a real-world analysis of the trend of recurrence and, consequently, the probability of survival in patients with BL/LA PDAC who underwent a pancreatectomy after neoadjuvant chemotherapy. The findings can facilitate data-driven discussions among physicians, patients, and families regarding the objectives of care. However, there is no consensus on the benefits of monitoring and the appropriate interval for monitoring these patients.

There is widespread variation in PDAC surveillance practices, with some societies and countries not advocating surveillance, presumably because of a lack of high-level evidence and a perceived lack of treatment options if recurrence is diagnosed [8]. The American Society of Clinical Oncology, French guidelines, and Japanese societies recommend CT imaging of the abdomen and pelvis every 3–6 months for the first two years after surgery and serum CA 19-9 monitoring in patients with elevated preoperative value [16].

The NCCN proposes surveillance with examination and clinical assessment for the presence of symptoms every 6 months for 2 years; however, the NCCN does not advocate radiographic imaging because data demonstrating its efficacy are lacking and does not specify the duration for which the follow-up should be continued [1]. The European Society for Medical Oncology (ESMO) proposes a program of follow-up prioritizing patient psychological state and willingness [17].

For surveillance to become widely adopted, there are two requirements: first, a reliable system to detect recurrence, avoiding useless and stressful examinations for patients; and second, a rational basis to support the treatment in these patients.

This study showed that with a 38% 5-year survival rate in the recent 11 years, survival is improving compared to that in earlier years (15% in a recent Netherlands Cancer Registry study from 2005 to 2016 [18]). Second, the peak risk of recurrence occurred during the first two postoperative years (44% in one year and 25% in the second), and the probability of recurrence then decreased with no recurrence after the seventh year. The risk factors involved in recurrence were identical to those previously reported in the literature, such as the CA 19-9 value at diagnosis, weight loss, tumor stage, and the number of positive lymph nodes [12,13,14,15,19,20]. Third, patients with T1/T2 N0 R0 had the lowest rate of recurrence within two postoperative years (19%), with no recurrence after this deadline.

Since the highest rate of recurrence occurred during the first two postoperative years, it might seem relevant to undertake consistent surveillance during the first two or three years at intervals of 3 months. The intervals should progressively widen to 6 months until the seventh year, and finally, every 12 months until the tenth year. This approach might allow the detection of recurrence at an asymptomatic stage, with the possibility of early secondary-line treatment; however, data are scarce regarding this [19,21,22].

There is strong evidence that patients with pancreatic cancer may benefit from chemotherapy. Randomized clinical trials have demonstrated that FOLFIRINOX is the most potent and effective treatment regimen in the metastatic and adjuvant settings. However, it remains unclear whether and to what extent the timing of treatment (i.e., neoadjuvant or adjuvant), number of cycles, and dose density are relevant to survival [23,24,25,26,27,28].

Concerning second-line chemotherapy, there is a clear need for well-designed trials for indications after FOLFIRINOX treatment failure. Gemcitabine combined with nab-paclitaxel offers some benefits [24]; however, the combination of platinum agents with gemcitabine or 5-fluorouracil (FU) remains the standard of care. However, the survival benefit provided by these combinations is limited and should be interpreted with caution, given the selection bias in this patient population [25].

In addition to the opportunity for systemic treatment, the importance of early detection of recurrence must be highlighted. At the time of recurrence, preserved performance status and isolated recurrence (local or distant, as opposed to regional or multiple-site recurrence) are independently associated with longer postresection overall survival [26]. Conversely, symptomatic recurrence is an independent predictor of poor postrecurrence survival [9]. Early detection of limited locoregional recurrence may also offer an opportunity for focal treatment. In an autopsy study, one-third of pancreatic cancer recurrences are isolated local recurrences [27,28], and iterative surgical resection [29] or local ablative therapy [30] could offer survival benefits. A previous study by Tjaden et al. demonstrated how the structured detection of recurrences facilitates the offering of subsequent treatments [31], and a randomized trial aimed at implementing surveillance in the same way in the Netherlands is ongoing [32]. On the other hand, Tzeng et al. demonstrated that with an incrementally increasing frequency of the surveillance with CA19-9 and CT scan every 6 months, the increase in costs has no associated survival benefits [11].

In addition, this analysis proposes different surveillance models according to the TNM and margin classification. Indeed, patients with T1/T2 N0R0 might follow a different model of surveillance, with straight monitoring for the first two years and less frequent control after that. These cases remain rare (18 patients in this series), and the available data suggest that patients prefer to be within a surveillance program despite having a good prognosis [33].

An important end point of cancer surveillance, apart from survival and risk of recurrence, is the quality of life. A crucial issue is whether CT should be performed only if signs and symptoms of recurrence are present or if it should be performed at regular intervals. Symptoms suggesting recurrence are usually abdominal pain, fatigue/weakness, back pain, weight loss, and nausea/loss of appetite. It may be difficult to avoid performing CT scans in patients after surgery for pancreatic cancer because such patients may frequently experience symptoms that would prompt diagnostic imaging. Furthermore, how the detection of recurrent disease and confronting asymptomatic pancreatic cancer patients with their dismal prognosis affects their quality of life remains to be established [9].

As patients with pancreatic cancer often seek more care than cure, reassurance through attending follow-up appointments and hearing from oncologists that they did not have a recurrence is important. Little is still known about the consequences of screening on the subjects’ emotional and psychological well-being. Many patients express the importance of timely access to clinicians or nurses at cancer centers to feel reassured during follow-up appointments [34]. Moreover, psychological symptoms in pancreatic cancer surveillance programs are considerable, and the burden is considerably higher than that reported for other cancers [35,36]. A population-based study demonstrated that after two years, 13% of patients with pancreatic cancer received their first hospital contact or first prescription of antidepressants [37,38]. Demoralization and feelings of a loss of control may be reasons for this finding; patients may experience greater depression if they perceive that they have very limited options with regard to medical treatment.

Regular follow-up with CT after surgery for PDAC is essential to identify tumor recurrence early in order to offer further disease-controlling measures or potentially curative options to patients. Nordby et al. demonstrated that both disease-free and overall survival were significantly better in asymptomatic patients. In such patients, the detection of disease through CT may facilitate patient eligibility for investigational studies or other forms of treatment. However, the patients have to be aware of the limitations of the detection of an asymptomatic recurrence and that no curative treatment is currently available [9].

In the end, the tripod of clinical examination, CA19-9, and CT scans remains the most valuable in terms of cost-effectiveness. However, some evidence suggests that PET-CT may be more effective than conventional CT in detecting PDAC recurrence, especially in differentiating pancreatic bed recurrence from normal postoperative changes [39,40]. Future predictive biomarkers, such as circulating tumor cells [41], will help to tailor the surveillance to the risk.

### 4.1. Strengths and Limitations

The strength of this study was the identification of a model of surveillance exploitable for all patients who underwent PDAC surgery after neoadjuvant chemotherapy, with no significant difference besides the well-known risk factors associated with recurrence. Our findings offer a contemporary and meaningful view of outcomes for patients with BL and LA PDAC after surgery, with an extended and “real-life” follow-up. Moreover, the findings could open up a discussion to tailorize the surveillance model for patients with favorable pathological findings with less straightforward monitoring.

Despite these strengths, this study has several limitations. First, there may be biases associated with its retrospective, single-institution design. Second, we associated BL and LA PDAC. This contestable choice was made for two reasons: for restaging after chemotherapy, with LA PDAC becoming BL after chemotherapy and for the simplification of the selection of the small sample of patients. Third, it does not evaluate the presence of symptoms at the recurrence diagnosis. Furthermore, it does not consider the utility of monitoring according to the treatment that can be realistically proposed for elderly people. Fourth, owing to the small number of patients, no conclusions could be drawn for patients receiving neoadjuvant chemoradiation. Finally, concerning the tumor response assessment, we did not measure the tumor size before and after chemotherapy to simplify the analysis of variable/complex factors and to focus on the global risk of recurrence. PDAC can present various radiological characteristics. Often, these tumors appear to be isointense and arduously measurable, and it can consequently be difficult to compare them at the re-evaluation scan or with the resected specimen. A dedicated study could focus on this variable in the future.

### 4.2. Next Generation

In this study, we confirmed the well-known risk factors associated with recurrence. However, in recent years, new predictive factors for recurrence and survival have been studied, which may contribute to patient management. The John Hopkins Hospital group recently proposed circulating tumor cells (CTCs) as biomarkers to predict survival in pancreatic cancer: in patients that were clinically disease-free 12 months postoperatively, CTC positivity was associated with higher rates of subsequent recurrence, and some patients demonstrated persistent CTCs postoperatively, which could represent minimal residual disease [41]. Another emerging biomarker is the immunoscore. Since cancer progression is strongly influenced by the host immune response, a T-lymphocyte-based immunoscore could be used as a putative biomarker to guide patient prognostication and management in pancreatic cancer [42,43]. The immunoscore was recently included in WHO and ESMO guidelines as the best clinical evidence in colon cancer, which is expected to refine the prognosis and thus adjust the chemotherapy decision-making process.

In addition, the study of postchemotherapy metabolic responses (PET) as surrogates of pathologic response might be another tool to suggest tumor activity and its correlation with the risk of recurrence and survival [19]. PET-CT is mostly used to monitor distant recurrence, ambiguous CT findings, and patients with normal CA19-9 levels. Using PET-CT as a complementary test, may lead to the underestimation of its diagnostic performance in detecting pancreatic cancer recurrence. In the absence of CT changes in tumor assessment and the evaluation of the metabolic response, PET-CT provides information on tumor viability. Thus, PET-CT is a potential critical standard radiologic examination for objective chemotherapeutic response assessment, establishing pretherapeutic avidity to compare posttreatment metabolic responses [44].

For the establishment of an efficient surveillance methodology, the above research tracks should be further investigated.

## 5. Conclusions

In conclusion, this study provided a real-world prognosis that encourages data-driven discussions among physicians, patients, and families during BL or LA PDAC treatment. Proposing a model for postoperative surveillance may facilitate standardization. The risk-benefit and cost-effectiveness of recurrence-focused surveillance remain unclear. Therefore, the potential side effects, including psychological harm and the economic costs of these examinations, must be considered. In future, there is a need to identify the appropriate level of correlation between efficient treatment and the likelihood of the most benefit from early detection of recurrent disease.

## Figures and Tables

**Table 1:** Baseline demographics of the 101 patients who underwent surgery after FOLFIRINOX.

Characteristics	
**Sex ratio** (M/F)	0.74
**Median age** (years, range)	64 (37–80)
**ASA score ≥ 3** (%)	16 (16)
**Mean BMI** (±SD), kg/m^2^	24.2 (±4.24)
**Weight loss > 5%** (%)	56 (55)
**Back pain** (%)	54 (53)
**Tumor localized in the head (%)**	71 (70)
**Biliary stenting** (%)	63 (62)
**Arterial invasion** (%)	31 (31)
**Locally advanced** (%)	6 (6)
Median CA 19-9 * (UI) (range)	152 (3–12100)
**Median number of cycles** (range)	6 (4–12)
**Additional CRT** (%)	8 (8)
**Surgery (%)**	
PD	69 (68)
TP	5 (5)
LP	27 (27)
**Vascular resection** (%)	67 (66)
**T3/T4 stage** (%)	67 (66)
**N+** (%)	62 (61)
**R0 resection** (%)	77 (76)
**Perineural invasion** (%)	78 (77)
**Recurrence** (%)	71 (70)

(ASA: American Society of Anesthesiologists; LA: locally advanced; BMI: body mass index; CA 19–9: carbohydrate antigen 19–9; CRT: chemoradiation; * at diagnosis and after jaundice resolution; PD: pancreaticoduodenectomy; TP: total pancreatectomy; LP, left pancreatectomy).

**Table 2:** Comparison of baseline, treatment, and histopathological characteristics between patients who did (R-group) or did not (NR-group) experience recurrence.

	R-Group	NR-Group	*p* Value
**n**	71	30	**-**
**Sex ratio** (M/F)	0.7	0.87	0.66
**Median age** (years, range)	64 (43–80)	63 (37–78)	1
**ASA score ≥ 3** (%)	13 (18)	3 (10)	0.38
**Mean BMI** (±SD), kg/m^2^	24.1 (±3.8)	24.2 (±4.8)	0.4
**Weight loss > 5%** (%)	22 (73)	34 (48)	**0.019**
**Back pain** (%)	36 (51)	17 (57)	0.58
**Tumor localized in the head (%)**	49 (69)	22 (73)	0.66
**Biliary stenting** (%)	45 (63)	18 (60)	0.75
**Arterial invasion** (%)	24(34)	6 (20)	0.2
**Locally advanced** (%)	5 (7)	1 (3.3)	0.66
Median CA 19-9 * (UI) (range)	420 (20–12100)	128 (3–1250)	**<0.01**
**Median number of cycles** (range)	6 (4–12)	4 (4–12)	0.47
**Adjuvant chemotherapy (%)**	47 (66)	19 (63)	0.78
**Additional CRT** (%)	6 (8.4)	2 (6.7)	1
**Vascular resection** (%)	48 (68)	19 (63)	0.81
**T3/T4 stage** (%)	54 (76)	13 (43)	**<0.01**
**N+** (%)	51 (72)	11 (37)	**<0.001**
**R0 resection** (%)	49 (69)	28 (93)	**0.01**
**Perineural invasion** (%)	59 (83)	19 (68)	0.095

(ASA: American Society of Anesthesiologists; LA: Locally Advanced; BMI: Body Mass Index; CA 19–9: Carbohydrate Antigen 19-9; CRT: chemoradiation; * at diagnosis and after jaundice resolution).

**Table 3:** Risk of recurrence according to pathologic findings.

	N0	N+
T1/T2	R1 100% R0 19% *	R1 100% R0 67%
T3/T4	R1 100% R0 64%	R1 86% R0 85%

* *p* = 0.023 compared to all other combinations. Green for lower risk of recurrence, orange for intermediate and red for higher risk of recurrence.

## Data Availability

The datasets generated and/or analyzed during the current study are not publicly available because of patient privacy concerns but are available from the corresponding author upon reasonable request.
